# Editorial: Molecular mechanisms of mental disorders: the contribution of genetic and environmental factors

**DOI:** 10.3389/fnmol.2026.1937671

**Published:** 2026-08-19

**Authors:** Martina Di Bartolomeo, Claudio D'Addario

**Affiliations:** 1 Department of Bioscience and Technology for Food, Agriculture and Environment, University of Teramo, Teramo, Italy; 2 Department of Clinical Neuroscience, Karolinska Institutet, Stockholm, Sweden

**Keywords:** epigenetics, gene environment interactions, mental disorders, molecular mechanisms, public health

To date mental disorders, including bipolar disorder (BD), schizophrenia (SCZ), and major depressive disorder (MDD), persist as 1 of the 10 primary contributors to the global burden of disease, showing no signs of a worldwide decline since 1990 (GBD 2019 Mental Disorders Collaborators, 2022). Mental health conditions involve a notable psychological disruption affecting a person's thinking, emotional control, or conduct. This state typically leads to substantial personal suffering or disrupts daily life activities (World Health Organization, 2022). Of note, mental disorders are usually the result of several contributing factors with a strong and dynamic gene-environment interplay (World Health Organization, 2022). A crucial point is the so-called “missing heritability”: genome-wide association studies (GWASs) explain only a small proportion of their heritability estimated by family studies (Gratten et al., 2014). This clearly indicates the importance of other factors such as the environmental ones and the role of epigenetic mechanisms (DNA methylation, histone modification, and miRNAs) in the gene-environment crosstalk molecular effects (Nestler et al., 2016).

The present Research Topic was conceived to clarify the complex architecture which characterized mental disorders bringing together preclinical and clinical contributions that highlight how genetic risk factors interact with environmental exposures at the molecular level, and how these interactions may be harnessed to develop more targeted, personalized therapeutic approaches.

The review of Ashvil et al. [Molecular interplay between glycogen synthase kinase 3 beta and A-kinase anchoring protein 11 in bipolar disorder: a narrative review] focuses on one of the most pressing clinical challenges in psychiatry: the identification of molecular targets in BD. In particular, this work aims to investigate the molecular interactions between AKAP11, a high-confidence risk gene which variants lead to seven-fold increased risk for BD and SCZ, and GSK-3β, widely considered the primary pharmacological target of lithium. Through the exploration of the molecular pathways where these proteins converge to modulate neurons and lithium's mode of action and the effects of the disruption of

this interplay by “edgetic” mutations, this paper provides a powerful demonstration of translating genetic discoveries into functional insights with immediate clinical relevance.

Epigenetic regulation takes center stage in the opinion article of Peedicayil and Santosh [Histone monoaminylation is a novel epigenetic mechanism in psychiatric disorders] which investigates a specific class of histone modifications, the covalent modification of histone proteins via the attachment of monoamines like serotonin, dopamine, and histamine, synthetizing emerging evidence for their role in psychiatric disorders. Considering that monoamine neurotransmission is both fundamentally targeted by psychiatric therapies and altered by environmental stress, this research unveils a conceptually novel dimension in the epigenetic underpinnings of mental illness.

Providing a complementary viewpoint, [Reported race-associated differences in control and schizophrenia post-mortem brain transcriptomes implicate stress-related and neuroimmune pathways] by Simmons et al. shows how specific molecular pathways are uniquely perturbed across race groups revealing multiple candidate pathways linked to SCZ within a race-specific context. These findings further support the inextricable link between biological and environmental drivers of gene expression, thereby demanding greater consideration of socio-environmental influences in the field of neurobiology.

The gene–environment interface is further explored at the level of neuroendocrine stress signaling in a mice model of MDD in the paper of Ueda et al. [Divergent and subnucleus-specific gene expression responses to chronic stress hormone exposure in the amygdala]. Mice exposed to chronic corticosterone show highly region-specific transcriptional programs within the amygdala, with distinct responses across subnuclei. These results underscore the necessity of spatially and anatomically resolved investigations to clarify the biological foundations of stress-induced psychiatric conditions like MDD, ultimately facilitating the development of precision therapeutic interventions.

Two papers explore the molecular mechanisms underlying distinct clinical diagnoses.

The review [Progress and challenges in exploring the pathogenesis of depressive disorders via RNA sequencing] by Zhang et al., delivers a current consolidation of transcriptomic perspectives on depressive disorders, examining the impact of RNA sequencing advancements—from bulk to single-nucleus resolution—to explore the pathogenesis of MDD, perinatal depression (PND), perimenopausal depression (PMD), and post-stroke depression (PSD). The authors highlight how these distinct clinical subtypes exhibit significant transcriptomic heterogeneity and encourage single-cell and spatial transcriptomic technologies to deconstruct tissue heterogeneity with the integration of multi-omics data to map causal regulatory networks.

Meanwhile, [The CB1R of mPFC is involved in anxiety-like behavior induced by 0.8/2.65 GHz dual-frequency electromagnetic radiation] by Sun et al. introduce electromagnetic field exposure as a novel environmental factor in molecular psychiatry, revealing that endocannabinoid signaling within the medial prefrontal cortex—specifically via CB1 receptor pathways—drives the anxiety-inducing effects of radiofrequency radiation in a rodent model. Consequently, this research extends the scope of environmental influences in psychiatric disorders and highlights the endocannabinoid network as a key convergence site for heterogenous external stressors.

Finally, Dong et al. in their review [Schizophrenia and type 2 diabetes risk: a systematic review and meta-analysis] contextualizes psychiatric disorders within the broader landscape of systemic physical comorbidities, mapping the bidirectional interplay between SCZ and metabolic dysfunction. These insights strengthen the premise that psychiatric illnesses cannot be isolated from systemic biology, highlighting that shared biological pathways (e.g., inflammatory, oxidative, and metabolic cascades) are responsible for the increased physical illness burden in patients with severe psychiatric diagnoses.

Taken together, these contributions illustrate both the breadth and the depth of current molecular psychiatric research, collectively advancing our understanding of how genetic architecture and environmental experience converge to produce mental illness. These insights also emphasize a critical directive for the future: research needs to combine multi-omic, longitudinal, and environmentally aware methodologies to resolve the persistent explanatory deficits that have historically constrained the discipline.

It is the Editors' hope that this Research Topic will inspire further research, leading to targeted therapeutic approaches that align with the distinct molecular individuality of each patient.
